# Influencing factors of community residents' pro-environmental behavior in East Dongting Lake National Nature Reserve under the policy intervention

**DOI:** 10.1038/s41598-023-32553-0

**Published:** 2023-04-13

**Authors:** Xianglong Tang, Jianqiong Yuan, Xitong Zeng

**Affiliations:** Central South University of Forestry and Technology, No. 498 Shaoshan South Road, Changsha, 410004 China

**Keywords:** Psychology and behaviour, Environmental impact, Sustainability

## Abstract

In the study of protected areas, the "Fences & fines" approach is increasingly becoming acknowledged as obsolete and ineffectual, and there is mounting evidence suggesting that the "Community-based conservation" approach is acquiring consideration. It is significant to identify which protection model or factors perform a definitive part in China. Taking the East Dongting Lake National Nature Reserve in China as a survey site, this paper utilizes semi-structured interviews and random questionnaires surveyed 431 households to investigate the relationship between "community-based conservation" approaches such aslegal system, ecological compensation, environmental education, community participation, concessions, livelihoods, job provision, intrinsic motivation and pro-environmental behavior. The regression results declare that intrinsic motivation (β = 0.390) and legal system (β = 0.212) are the most effective factors impacting on pro-environmental behavior; concessions has a negative conflict on preservation;but other "community-based conservation" approaches had insignificant positive impacts on pro-environmental behavior. Further mediating effects analysis indicated that intrinsic motivation (B = 0.3899, t = 11.9694, p < 0.01) mediates between legal system and pro-environmental behavior of community residents, legal system promotes pro-environmental behavior by promoting intrinsic motivation, which is more effective than legal system promoting pro-environmental behavior directly. This demonstrates that “Fence and fine approach" still is an effective management tool which can shape community residents' positive attitude towards conservation and pro-environmental behavior especially protected areas with large communities. And appropriate "community-based conservation" approaches can mitigate conflicts between special groups with the combination of these two approaches, the management of protected areas can be successful. This supplies a valuable real-world case for the current debate on conservation and improved human livelihoods.

## Introduction

In the discussion on biodiversity preservation, two models are perceived to exist: outright preservation and practical use. One model uses top-down conservation management, using legal means (based on natural sciences). Numerous studies have shown that this approach can cause disagreements between regulations and members of the local community^1–4^, has special consequences for the livelihoods of the local community residents^5^. Another model uses an approach involving community participation and multidisciplinary management to achieve sustainable use of resources^6^. Thus we need novel approaches and community support for conservation, but community development aspirations may conflict with conservation objectives. This makes the implementation of this approach exceedingly challenging^7^. Years of research have shown that in most cases, a "top-down" approach to protection does not achieve the desired goals^5^,Residents' interests are disregarded, conflicts between humans and environment arise as a result, and local sustainability is heavily impacted^8^. Currently, the world has reversed to the second approach—a "community-based conservation" approach. Governments distribute power and responsibility among local resource users to relegate the cost of biodiversity reserves to local people^9^.

In the practice of the "community-based conservation" approach, biodiversity conservationists all over the planet have created numerous inventive means to regulate the biological protection conduct of local area occupants. These means incorporate alternative livelihoods, environmental education, community participation, job provision, ecological compensation, etc. For instance, in some developing countries and impoverished areas, the consumption and trade of wildlife is minimized by supplying hunters with alternative sources of protein and income generation^10,11^; enriching community residents' income sources and optimizing household livelihood structures by supplying ecological compensation, job opportunities and vocational training^12–16^. However, the effectiveness of these means is still controversial.

Simultaneously, the "Fence & fine" approach has not been abandoned completely. The conservation of biodiversity requires a comprehensive system of laws, regulations and policies to guarantee the execution of related exercises^17^. Both hard and soft laws are acknowledged as key means for protecting biodiversity^18^. Most countries have enacted laws on the protection of natural assets such as organisms, forests, wetlands, and established national systems of protected areas to confine human activities as a mean to secure explicit biological species^19,20^. However during the protection strategy advancement process, destructive human activities were utterly impermissible, furthermore, the interests of local communities occasionally included in the management of protected areas^21^.Subsequently, local residents frequently possess threat toward protected areas^17^. The "Fence & fine" approach has likewise been progressively condemn by scholars.

In the 66-year history of biodiversity conservation and ecosystem maintenance in China^22^. The Chinese government has always adopted the "Fence & fine" approach^23^, and aided some community participation programs such as alternative livelihood, ecological compensation, environmental education to promote local people's pro-environmental behavior (PEB) and further develop the protection effect^24–26^. Evidence testifies that with the implementation of this set of policy means, China has generated incredible accomplishments in biodiversity conservation and ecosystem restoration^17,27,28^.

The PEB of community residents is a vital component in the achievement of protected areas. Few studies have comprehensively assessed the impact of various conservation models of policy interventions on PEB of community residents. PEB is significantly influenced by an accumulation of factors such as livelihood status, willingness to protect, and psychological compnents^29–31^. A more comprehensive study is required to ascertain whether PEB of community residents arises in the management of communal protected areas in China, which factors ascertain PEB of community residents, and which factors play a crucial function in conservation.

This study sought to compare the impact of various policy means used by the Chinese government on PEB of community residents and to explore the determinant factors of PEB in protected areas. These factors are household livelihoods, ecological compensation, concessions, job provision, environmental education, community participation and legal system. They have been consistently demonstrated to have a significant effect on PEB. Simultaneously, in order to discover the initiative of community residents towards protection, the measurement of intrinsic motivation was included. Compared to preceding studies in the field of environmental management, it is a novel attempt to compare all policy interventions together in this "top-down" form of conservation in China. The main objectives of this study are as follows: (1) What factors influence the PEB of community residents? (2) Is there an intrinsic motivation of community residents to protect? (3) What is the function relationship between policy interventions, intrinsic motivation, and PEB?

## Materials and methods

### Study site

**Figure 1:**
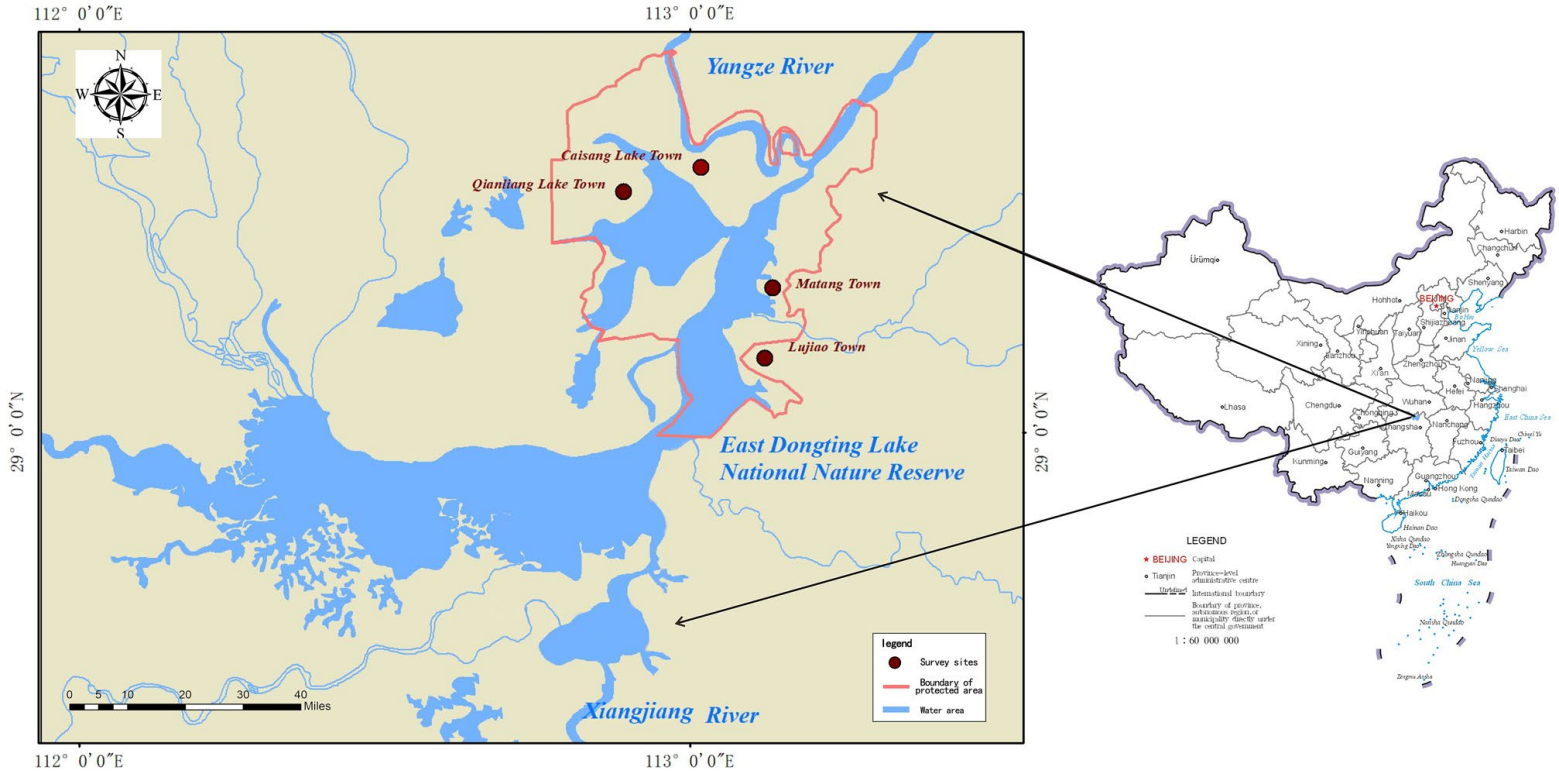
Study area: East Dongting Lake National Nature Reserve; Brown dots show four investigation points and the red line shows the boundary of the protected areas. (Created with ArcGIS Pro3.02; https://www.esri.com/en-us/arcgis/products/arcgis-pro/overview).

East Dongting Lake National Nature Reserve (EDT), located in Hunan Province in China, is a Ramsar site and one of the eco-regions that listed in the Global 200 as the most crucial to the conservation of global biodiversity. EDT also has been recognized as the key wintering region in Yangtze River floodplain for hundreds of thousands of migratory waterbirds of the East Asian–Australasian Flyway. (The location of the study area is shown in Fig. 1).

During the 40 years of conservation, EDT has adopted a series of conservation means to protect the wetlands. Some are geared toward the natural environment, such as wetland restoration, species monitoring, and wildlife rescue. Some are geared towards community residents, such as ecological migration, introduction of laws, fishing and sand bans, etc. The implementation of these protection measures has achieved great success. In the synchronized survey of wintering waterbirds from January 11 to 12, 2021, a total of more than 288,000 wintering waterbirds of 53 species were monitored, with the number maintaining an increase for the fourth consecutive year. The conservation of EDT in terms of biodiversity and wetland ecology has been successful, but it remains to be seen whether the management of community residents has been equally successful.

### Data collection and sampling strategy

Four Towns around the EDT were selected as survey sites: Caisang Lake Town, Qianliang Lake Town, Matang Town, and Lujiao Town, from 6 to 10 January and 4–12 May 2022, we surveyed the local people, including former fishermen, farmers, ecotourism practitioners, and reserve managers. This research is supported by the research institute, and all methods were carried out in accordance with relevant guidelines and regulations. All surveys were communicated to respondents in writing or verbally, and respondents consent was obtained.

First is a total of 5 times group meeting discussion. The first meeting was held at the East Dongting Lake National Nature Reserve Administration, with six participants. The research team investigated the conservation status and challenges of EDT, and generated reasonable inquiries about the questionnaire and survey sites. The second meeting was held at the Qianliang Lake Town Community Management Committee office, with a total of five participants, to confer with the village chief on the relationship between the reserve and village development. The third, fourth and fifth meetings were held at the homes of villagers in Caisanghu Town, Matang Town, and Lujiao Town respectively, with a total of 16 participants. The research team communicated with villagers about the management policies of the reserve, their willingness to protect as well as their personal living conditions. Subsequently, we conducted semi-structured interviews with the four village leaders and the dean of the east dongting lake.

Lastly, on a random sampling, a total of 431questionnairs were completed, including 88 households in Caisang Lake town, 122 households in Qianlianghu town, 123 households in Matang town, and 98 households in Lujiao town. To alleviate data collection and personal interviews, three master's students and one undergraduate student in forestry were recruited as research assistants, and a local person was contacted as a guide.

### Measures and indicator

The questionnaire incorporated four parts. The first part contains the socio-demographic profile of the respondents (Table 1). The second part investigates the extent to which livelihood status, legal system, environmental education, community participation, ecological compensation, job provision, and concessions affect residents. For the livelihoods survey, we did not use the Sustainable Livelihoods Analysis Framework of the Department for International Development (DFID). It is difficult for farmers to count the real data. We directly inquired about the family's income; regarding the impact of legal system on residents, four questions were used to measure the four dimensions of law learning, law knowledge, law compliance and law usage with reference to related studies^32,33^; regarding ecological compensation, we calculated the amount of ecological compensation received by community residents; the job opportunities in this paper allude to the public service jobs supplied by the government regarding ecological conservation. These public service jobs can aid community residents accomplish their livelihood transition^34^. Engaging in nature conservation-related work will increase the PEB of residents^29^. We accomplished this aspect of the survey by investigating the relevance of occupation to nature conservation and the length of occupation; there is an extraordinary arrangement of research on community involvement, and we accomplished the survey with three questions in three dimensions: conservation involvement, planning involvement, and monitoring involvement^35–37^; with respect to the survey on environmental education, we assessed community residents' participation in environmental education activities by three questions in two dimensions: environmental knowledge and channels of environmental education^35,36^; concessions are non-resource-consuming operations under government supervision for a specified period, scope and volume, as authorized by the protected area manager in accordance with the law^37^. We directly calculated the income of farmers who carried out concession projects. The third and fourth part concern with the protection of intrinsic motivation and PEB measurements. The two-part scale was developed with reference to the results of preceding studies and the genuine situation of the survey sites^30,38–41^. All measurements were generated using the Likert scale.

**Table 1:** Characteristics of the sample (N = 431).

Characteristics	Percentage (%)
Sex	
Male	51.97
Female	48.03
Age	
16–25	9.98
26–35	11.60
36–45	16.71
46–60	38.98
> 60	22.74
Family income (per year)	
≤ 300,000	30.39
30,001–50,000	32.95
50,001–80,000	22.27
80,001–100,000	9. 05
> 100,000	5.34
Education	
Primary school	32.715
Junior high school	36.66
Senior high school	25.06
University or Junior College	5.57

### Data analysis

First, the reliability and validity of the scale were measured with SPSS 20.0, The Kaiser–Meyer–Olkin score is 0.853, and Cronbach's alpha is 0.907. Subsequently, mean scores were calculated for each variable prepared for the quantitative study, and stepwise multiple linear regression analysis was performed. The predictor variables were livelihood status, community participation, environmental education, legal system, intrinsic motivation, job provision, concessions, and ecological compensation. The significant influences among them were selected to explore the PEB influence paths among the predictors.

### Informed consent

Informed consent was obtained from all subjects involved in the study.

### Research approval

The research is approved by Academic Committee of College of Tourism, Central South University of Forestry and Technology, and all methods were carried out in accordance with relevant guidelines and regulations. All surveys were communicated to respondents in writing or verbally, and respondents consent was obtained.

## Results

### The function of predicting pro-environmental behavior

Table 2 shows the process of this stepwise regression model. It can be seen that with the gradual introduction of independent variables, the fit of the model increases until the last independent variable enters, the model cannot enter new independent variables, and the operation stops. Model 3 is the final model with a fit of 44.2%, implying that the three independent variables introduced by the stepwise regression model explain 44.2% of the "PEB".

**Table 2:** Multiple linear regression model for pro-environmental behavior.

Model	Model 1		Model 2		Model 3	
	β	Sig	β	Sig	β	Sig
Constant	2.100	0.000***	1.631	0.000***	1.762	0.000***
Intrinsic motivation	0.479	0.000***	0.383	0.000***	0.390	0.000***
Legal system			0.219	0.000***	0.212	0.000***
Concession					− 0.086	0.000***
Livelihood status					0.030	0.420
Community participation					0.045	0.242
Environmental education					0.023	0.556
Job provision					0.054	0.885
Ecological compensation					0.047	0.082
Sig		0.000		0.000		0.000
R		0.623		0.655		0.668
R^2^		0.386		0.427		0.442
Adjusted R^2^		0.520		0.502		0.496

***p < 0.001.

In conclusion from Table 2 Model 3, livelihood status (Sig. = 0.420), community participation (Sig. = 0.242), environmental education (Sig. = 0.556), job opportunities (Sig. = 0.885) and ecological compensation (Sig. = 0.082) are all greater than 0.05, have no significant impact on PEB, and are eliminated in the final model.

**Figure 2:**
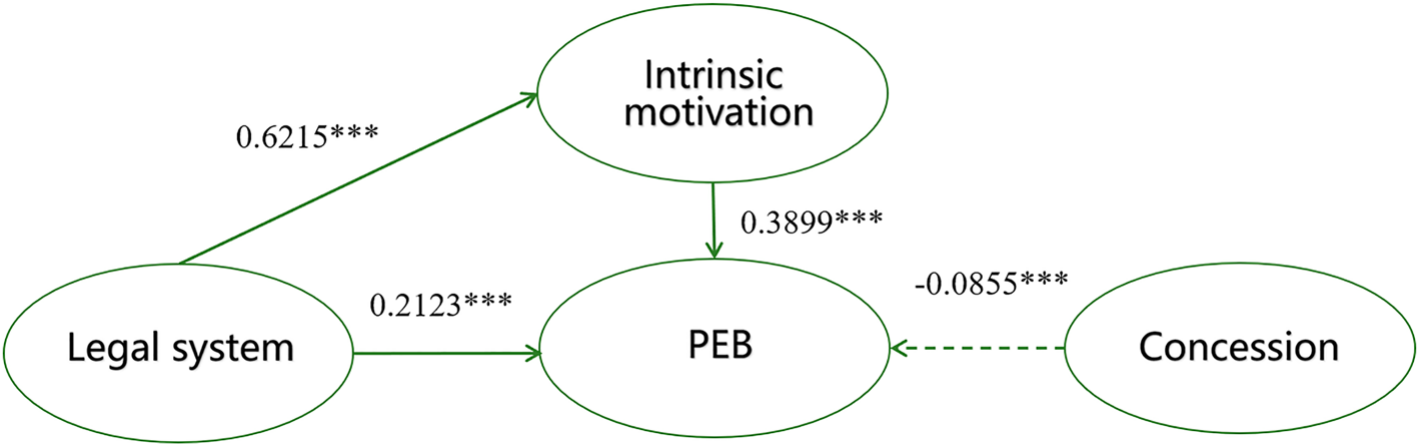
A relationship model of intrinsic motivation, legal system, concession and pro-environmental behavior.

From Table 4 Model 3, it can be seen that intrinsic motivation (β = 0.390) and legal system (β = 0.212) have a significant positive effect on PEB, and concession (β = -0.086) has a significant destructive effect on PEB. The standardized regression coefficient for intrinsic motivation is 0.390(> 0). This means that a 1-point increase in "intrinsic motivation" leads to a 0.390-point increase in "PEB". "Concession” is − 0.086(< 0), meaning that a 1-point increase in "Concession" can result in a − 0.086-point decrease in "PEB". (The curve graph of factors are shown in Fig. 2).

There is no multiple correlation among the three independent variables, and the VIFs are all less than 5, which means that the results of this operation are reliable. Based on all the above analyses, the regression equation between the independent and dependent variables was derived as follows:

$${\text{PEB}} = {1}.{762} + 0.{39}0*{\text{Intrinsic motivation}} + 0.{212}*{\text{Legal system}} - 0.0{86}*{\text{Concession}}$$

### The mediation function of intrinsic motivation in the relationship between legal system and pro-environmental behavior

To test whether there is a mediating function of intrinsic motivation in the legal system and PEB, Bootstrap test was conducted using SPSS Process. First, Model4 in SPSS macro (Model 4 is a simple mediation model), while controlling for the concessions, was used to test the mediation effect of intrinsic motivation in the relationship between the legal system and PEB. The results (see Table 3) show that the predictive effect of legal system on PEB was significant (B = 0.4546, t = 11.9675, p < 0.01), and the direct predictive effect of legal system on PEB remained significant when mediating variables were included, (B = 0.2123, t = 5.4951, p < 0.01); the predictive effect of legal system on intrinsic motivation was significant (B = 0.6215, t = 12.7256, p < 0.01), as was the predictive effect of intrinsic motivation on PEB (B = 0.6215, t = 12.7256, p < 0.01). In addition, the upper and lower limits of the bootstrap 95% confidence intervals for the direct effect of legal system on PEB and the mediating effect of intrinsic motivation did not contain 0 (see Table 4). This suggests that the legal system is able to predict PEB not only directly, but also through the mediating effect of intrinsic motivation (see Fig. 2). This direct effect (0.2423) and mediating effect (0.2123) accounted for 53.3.7% and 46.70% of the total effect (0.5503), respectively.

**Table 3:** Testing and effect analysis of Intrinsic motivate on mediational model.

Result variable	Predicting variable	R^2^	F	B	t	p
PEB		0.6679	114.6462			
	Concession			− 0.0855	− 3.5712	0.0004***
	Legal system			0.2123	5.4951	0***
	Intrinsic motivation			0.3899	11.9694	0***
PEB		0.5102	75.3048			
	Concession			− 0.0687	− 2.4883	0.0132**
	Legal system			0.4546	11.9675	0***
Intrinsic motivation		0.525	81.4452			
	Concession			0.0432	1.2188	0***
	Legal system			0.6215	12.7256	0***

**p < 0.05.

***p < 0.001.

**Table 4:** Analysis of the mediation function of intrinsic motivation on legal system and pro-environmental behavior.

	Effect	BootSE	BootLLCI	BootULCI	Proportion (%)
Indirect effect	0.2423	0.0282	0.1901	0.3011	53.3
Direct effect	0.2123	0.0386	0.1364	0.2883	46.7
Total effect	0.4546	0.0403	0.3751	0.5336	

## Discussion

### How do law plays a key function in nature protection

In the study, legalism (β = 0.212) was found to be a determinant in shaping the PEB of protected area residents. This is basically the same as what Zhengzao Wang and Xianqiang Mao found in China's Sanjiangyuan National Park, where command and control policies impose directly influences on natives' behavior^26^. In The Dominican Republic, George Holmes found that conservation of the Ebano Verde Scientific Reserve can in fact be successful when controlled by law, despite the fact that local residents failed to support conservation^42^. In EDT, additionally to the Environmental Protection Law of the People's Republic of China, the Forest Law, Grassland Law, Law on Wildlife Protection and Nature Reserve Regulations, the local government has promulgated two regional regulations: the Regulations on the Protection of Dongting Lake in Hunan Province and the Regulations on the East Dongting Lake National Nature Reserve in Yueyang City. A highly-developed system of laws and regulations supplies elaborated and rigorous rules for the behavior of community residents. This eradicate any plausible anthropogenic nature-damaging activities and directly formulate the pro-environmental behavior of the residents in relation to biodiversity.

Likewise, the improvement of the local non-agricultural economy has aided local residents to reduce their dependence on the natural resources of the protected area, and supplied a direct material premise to the execution of the law. In 2021, the per capita disposable income of farmers in the region was 19,752 RMB, well above the international poverty line of 5329 RMB (latest exchange rate: 1 RMB = 0.1503 USD) and the national per capita disposable income of 18,931 RMB that year. Fishermen who make their living from the natural resources of the reserve have moved to new settlements. The government has supplied them with compensation funds, new resettlement, and supplied vocational training, and they could easily find an alternative way to make a living in the urban center.

### How do intrinsic protection motives arise?

The study found that intrinsic motivation of residents (β = 0.390) was another dominant component in shaping PEB. This is in accordance with the findings of Cetas^43^. This demonstrates that the willingness and initiative of community residents to protect has reached an unprecedented height in the EDT. The result shows that the means of the intrinsic motivation of local residents was 3.8, with a total value of 5. The Local residents' intrinsic protection motives has a better performance. There is not apparent conflict with the protected areas or that conflict has been resolved. In our interviews with community residents, we also found that they all support the protection of protected areas. While there is still some complaints, such as restrictions on fishing and the loss of crops due to birds, this will not fundamentally change their support for the reserve. This might come from the expanded ecological awareness with Chinese people. In the 2019 Chinese Citizens' Ecological and Environmental Behavior Survey Report published by the Chinese Ministry of Ecology and Environment, the Chinese citizen public generally has an awareness of environmental responsibility and a strong willingness to behave environmentally friendly. About 90% of the respondents can basically "avoid eating terrestrial wild animals" or "refuse to buy rare wildlife products such as fur, bone products and pharmaceuticals"^44^. In a study on the willingness to pay for ecological conservation among the residents of EDT communities, 84.7% of the respondents were willing to supply some financial remuneration to the ecological service providers of EDT as a beneficiary^45^.

### The mediation function of intrinsic motivation between legal system and pro-environmental behavior

Intrinsic motivation had a mediating effect in (B = 0.3899, t = 11.9694, p < 0.01) legal system and PEB. The legal system not merely directly shapes PEB, but also enhances PEB by enhancing community residents' intrinsic motivation, which is more strongly expressed than legal system directly enhancing community residents' PEB. This is very consistent with the interviews of some residents. They are familiar with the laws and policies of the protected area and will actively sustain the effectiveness of the laws. This supports the study of Cai^46^ and Bertoldo^47,48^, that legal laws regulate the relationship between human and nature. Since human behavior occurs in time and space, such laws take effect at a certain time and space (land). As time extrapolates, the binding force of the legal system will be transformed from mandatory formal laws to informal areas, resulting in generally accepted beliefs and behaviors in society. This phenomenon has been verified in some EU countries such as France, the UK^49^ and Portugal^50^. It can be concluded that pro-environmental beliefs and behaviors, under the influence of the rule of law, are becoming a universal social value in EDT.

### Community-based approach foster small parts of specific households

In this survey, household livelihood status (β = 0.03, Sig = 0.420), ecological compensation (β = 0.047, Sig = 0.193), job provision (β = 0.054, Sig = 0.156), environmental education (β = 0.023, Sig = 0.556), and community participation (β = 0.045, Sig = 0.242) did not have a significant impact on PEB, this is not a single case.

In a meta-analysis of 21 alternative livelihood projects, only 9 projects were reported to be effective in improving local conservation attitudes, reducing environmental damage, and achieving improvements in biodiversity conservation goals^51^. These alternative livelihoods are implemented primarily to address the destructive impacts of conservation on impoverished communities^52^. The EDT region now is not an impoverished region, nevertheless a developed industrial area with a GDP per capita of $13,518 (2021). Working in the urban center constitutes a large part of the family's livelihood. The survey shows that 76.188% of the households are independent on EDT for their livelihood, and the improvement of the environment will improve their quality of life, so they support ecological protection regardless of the household's livelihood status.

Ecological compensation had insignificant effect on the PEB of community residents in the area. Ecological compensation are rare in developing countries, but some regions are experimenting^53^. Land in China is owned by the state, and direct payments in EDT are only for former fishermen who have given up fishing. Fishermen are able to receive fees on fishing gear sold to government, pensions (60,000 rmb/person), resettlement houses by the government, etc. to compensate for their loss in giving up fishing. For most other community residents, they do not acquire ecological compensation, and thus, ecological compensation only plays an insignificant positive function in shaping the PEB of the community residents in the EDT.

Direct job opportunities are also rare in the EDT, and the Reserve Authority can only offer a small number of positions as lake patrollers. Green experiences increase PEB^54^. In the interviews with the lake patrollers ' families, their environmental protection awareness and behavior are generally higher than the average level. This part of the population is less than 20 households, so that the job supply only plays an insignificant positive function in the whole EDT.

In the field survey, it was found that EDT did not attempt much environmental education work, they rarely organized environmental education activities and only installed a diminutive number of publicity signs in some specific areas. Interviews discovered that community residents were aware of the existence of these publicity signs, but lacked interest in their content. The inadequate provision of environmental education and the lack of interest of community residents have resulted in limited impact of environmental education on residents' PEB.

Stepwise multiple linear regression results show that community participation plays a non-significant positive function on PEB. It is due to the lack of community participation, Cetas and Yasué had the same finding^43^. The population around the EDT is so densely that it is impractical to invite every household to participate in the management of the reserve.

Little community participation is manifested in the governance of EDT. These community-based approaches only target a few specific groups, mainly fishermen, and have shown positive effects in shaping their PEB. The ownership of the land belongs to the country and this directly contributes to the low participation of co-management, this is a common phenomenon in China^55^.

Community participation is important, it should considered not as a panacea^56^. They have had limited success in livelihoods, decentralization and sustainability^57^. But we can negate the function played by a community-based approach. The EDT does not just rely on "Fences & fines", but a whole set of methods to promote the conservation behavior of community residents, such as ecological compensation, laws and regulations, education, community participation and alternative livelihoods, are implemented. These methods have largely eased the conflicts between some special community residents, such as fishermen, and conservation. From a more comprehensive perspective, both “Community-based conservation” and “Fences & fines” are needed, they play different functions, one is Bad cop, and the other is Good cop, they play different functions in different biodiversity geographical area.

### Nonideal effects of concession

Concessions is one of the effective ways to guarantee the successful transformation of livelihoods of residents who are affected by ecological protection, and to alleviate community conflicts and prevent community conflicts^58^. It has also been shown that this approach encourages conservation behavior and contributes to achieving sustainable utilization of resources^31,59^. However, there is no such discovery in the EDT. The concession project of the EDT is mainly crayfish farming. These crayfish have become an ideal food for birds. The more crayfish are bred, the greater the loss of farmers, which affected their PEB. Not all concession projects are conducive to mitigating the tensions between community development and conservation, and many novel sustainable solutions may fail in another location^51^. In-depth empirical research in any area is a necessary process^5^.

## Conclusions and limitations

### Conclusions

“Fence and fine approach" still is an effective management tool, especially with large local communities. Nowadays, this approach is gradually being abandoned by conservationists due to its inability to effectively achieve conservation goals^60^. However, through long-term strict legal management, community residents have increased their conservation intrinsic motivation and generated pro-environmental behaviors that ultimately contribute to the success of nature reserve management in China. Therefore, the "fence and fine approach" still plays an important role in the management of communal protected areas.

"Community-based conservation" approaches should be adjust to protected areas with large local communities. "Community-based conservation" approaches were applied to special groups and not play a major function in EDT. With more and more conservators embracing community-based approaches, we need to think carefully and apply them wisely according to local realities and more innovation is needed in sustainable industrial development, missionary approaches, and public participation. For example, environment education can reach more local people through Digital Media, birdwatching but not crayfish farming will not arise new conflict etc.

In summary, through our investigation of the East Dongting Lake National Reserve, we found that the management of nature reserves in China can be successful through a long-term strict legal management model and an appropriate "community-based conservation" approach. This is a successful experience in biodiversity conservation in China. It supplies a valuable real-world example for the current debate on conservation and improved human livelihoods.

### Limitations

The results of this regression analysis demonstrate that intrinsic motivation, legal system and concessions can explain 44.2% of PEB, which means that there are still some influencing factors that need to be explored, and these factors are more likely to come from individuals, such as place attachment^30^, It may also come from societies such as social norms^47^, which require more case studies.

## Data Availability

The datasets generated and analysed during the current study are not publicly available due to the confidentiality of the follow-up study, but are available from the corresponding author on reasonable request.
